# Effects of oral, smoked, and vaporized cannabis on endocrine pathways related to appetite and metabolism: a randomized, double-blind, placebo-controlled, human laboratory study

**DOI:** 10.1038/s41398-020-0756-3

**Published:** 2020-02-19

**Authors:** Mehdi Farokhnia, Gray R. McDiarmid, Matthew N. Newmeyer, Vikas Munjal, Osama A. Abulseoud, Marilyn A. Huestis, Lorenzo Leggio

**Affiliations:** 1 Clinical Psychoneuroendocrinology and Neuropsychopharmacology Section, National Institute on Drug Abuse Intramural Research Program and National Institute on Alcohol Abuse and Alcoholism Division of Intramural Clinical and Biological Research, National Institutes of Health, Baltimore and Bethesda, MD USA; 2 Center on Compulsive Behaviors, National Institutes of Health, Bethesda, MD USA; 3 Johns Hopkins Bloomberg School of Public Health, Johns Hopkins University, Baltimore, MD USA; 4 Chemistry and Drug Metabolism Section, National Institute on Drug Abuse Intramural Research Program, National Institutes of Health, Baltimore, MD USA; 5 Lambert Center for the Study of Medicinal Cannabis and Hemp, Thomas Jefferson University, Philadelphia, PA USA; 6 Medication Development Program, National Institute on Drug Abuse Intramural Research Program, National Institutes of Health, Baltimore, MD USA; 7 Center for Alcohol and Addiction Studies, Department of Behavioral and Social Sciences, Brown University, Providence, RI USA

**Keywords:** Addiction, Physiology

## Abstract

As perspectives on cannabis continue to shift, understanding the physiological and behavioral effects of cannabis use is of paramount importance. Previous data suggest that cannabis use influences food intake, appetite, and metabolism, yet human research in this regard remains scant. The present study investigated the effects of cannabis administration, via different routes, on peripheral concentrations of appetitive and metabolic hormones in a sample of cannabis users. This was a randomized, crossover, double-blind, placebo-controlled study. Twenty participants underwent four experimental sessions during which oral cannabis, smoked cannabis, vaporized cannabis, or placebo was administered. Active compounds contained 6.9 ± 0.95% (~50.6 mg) ∆9-tetrahydrocannabinol (THC). Repeated blood samples were obtained, and the following endocrine markers were measured: total ghrelin, acyl-ghrelin, leptin, glucagon-like peptide-1 (GLP-1), and insulin. Results showed a significant drug main effect (*p* = 0.001), as well as a significant drug × time-point interaction effect (*p* = 0.01) on insulin. The spike in blood insulin concentrations observed under the placebo condition (probably due to the intake of brownie) was blunted by cannabis administration. A significant drug main effect (*p* = 0.001), as well as a trend-level drug × time-point interaction effect (*p* = 0.08) was also detected for GLP-1, suggesting that GLP-1 concentrations were lower under cannabis, compared to the placebo condition. Finally, a significant drug main effect (*p* = 0.01) was found for total ghrelin, suggesting that total ghrelin concentrations during the oral cannabis session were higher than the smoked and vaporized cannabis sessions. In conclusion, cannabis administration in this study modulated blood concentrations of some appetitive and metabolic hormones, chiefly insulin, in cannabis users. Understanding the mechanisms underpinning these effects may provide additional information on the cross-talk between cannabinoids and physiological pathways related to appetite and metabolism.

## Introduction

Perspectives on cannabis (marijuana) use are shifting throughout the world, politically and scientifically. According to the United Nations Office on Drugs and Crime, cannabis is the most commonly cultivated, trafficked, and used illicit drug worldwide, with an estimated 192.2 million users (3.9% of the global population)^1^. With cannabis medicalization and legalization increasing, the prevalence of cannabis use, in various forms, is also predicted to increase^2,3^. Several studies indicate that recreational cannabis use can have detrimental effects on physical and mental health^4–7^. On the other hand, continued work on cannabinoids for medical use resulted in the Food and Drug Administration’s (FDA) approval of cannabidiol (CBD), in the form of Epidiolex, for treating rare and severe forms of epilepsy, and of synthetic ∆9-9 tetrahydrocannabinol (THC), dronabinol, and nabilone, for preventing nausea and vomiting associated with chemotherapy. Accordingly, understanding the physiological and behavioral effects of cannabis use, recreationally or medicinally, is of paramount importance.

Scientific knowledge about the pharmacological actions of exogenous cannabinoids was considerably informed by the discovery of the G protein-coupled cannabinoid receptors (CB_1_ and CB_2_) and their endogenous ligands known as endocannabinoids (anandamide and 2-arachidonoylglycerol (2-AG)) which, along with their synthesis/degradation enzymes, collectively constitute the endocannabinoid system. Upon production and release, endocannabinoids bind to cannabinoid receptors located on pre-synaptic membranes and change neuronal excitability by modulating the release of different neurotransmitters^8–11^. Cannabinoid receptors are expressed not only in the brain, but also in the gut and other peripheral organs involved in food intake, metabolism, and energy homeostasis^12–14^. Previous studies suggest that agonism of the cannabinoid receptors, by either endocannabinoids or exogenous cannabinoids, acutely stimulates food craving, intake, and reward, and promotes the storage of energy in adipose tissues, whereas antagonism of the cannabinoid receptors reduces food intake and body weight^15–19^. On the other hand, chronic daily administration of THC suppresses weight gain, fat mass gain, and caloric intake in diet-induced, obese rats^20^, and epidemiological data indicate an association between chronic cannabis use and decreased prevalence of obesity and diabetes^21–26^. These findings suggest that cannabinoids play important roles in modulating appetitive behaviors and metabolic processes. However, more studies are required to shed light on the complexity of this cross-talk as, for example, acute versus chronic cannabis use may lead to different, and even opposite, outcomes^27^.

The hypothalamus plays a major role in food seeking and consummatory behaviors, representing a central hub for regulating appetite, metabolism, and energy homeostasis. Specifically, the hypothalamus controls homeostatic feeding, communicates with the mesolimbic system to modulate hedonic feeding, and interacts with peripheral organs to regulate endocrine pathways involved in hunger and satiety^28,29^. Several hypothalamic orexigenic and anorexigenic pathways are influenced by the endocannabinoid system^30,31^. The majority of previous evidence suggest that orexigenic actions of cannabinoids are linked to CB_1_ receptors located in the hypothalamus^32–34^, with some research also implicating the role of CB_2_ receptors in increased food consumption^35^. A close relationship also exists between the endocannabinoid system and endocrine pathways involved in metabolic regulation and food seeking behaviors^36–39^. For example, investigators found that orexigenic effects of systemically administered ghrelin (also known as the “hunger hormone”) were abolished in CB_1_ receptor knockout mice^40^, and pharmacological blockade of the CB_1_ receptor, via rimonabant administration, attenuated ghrelin-induced activation of the mesolimbic dopamine system – a key pathway involved in reward processing^41^. Activation of cannabinoid receptors, on the other hand, enhances leptin sensitivity^42,43^ and inhibits insulin secretion and insulin receptor signaling^44,45^. Hypothetically, the link between cannabis use and these endocrine pathways may be bidirectional, as growing evidence suggests that appetitive and metabolic hormones may play mechanistic roles in the development and progression of drug seeking behaviors^46,47^.

While previous data point to a close link between cannabis use and energy homeostasis, clinical research in this regard remains scant. In the only published human study, to our knowledge, looking at appetitive and metabolic hormones, smoked medicinal cannabis (as a treatment for neuropathic pain) was tested in adult men positive for human immunodeficiency virus (HIV). In this pilot, crossover, double-blind study, cannabis administration increased blood concentrations of ghrelin and leptin, decreased peptide YY (PYY) concentrations, and had no significant effects on insulin^48^. The goal of the present study was to explore the effects of cannabis administration on peripheral concentrations of endocrine markers related to appetite and metabolism in a sample of cannabis users and to build the foundation for future studies in this regard. This study also aimed at exploring potential differences in the effects of cannabis on endocrine outcomes when administered via different routes. There are at least two reasons why this additional aim is important. First, the route of cannabis administration (oral, smoked, or vaporized) alters THC pharmacokinetics, as well as its cardiorespiratory and subjective effects^49–52^. Second, cannabis legalization has led to an increase in the rates of oral and vaporized use, as compared to smoking^53^. Therefore, not only does assessing common routes of cannabis use provide information relevant to real-world conditions but may also elaborate on the general cross-talk between cannabis and appetitive/metabolic pathways.

## Materials and methods

### Study design, participants, and procedures

This was a randomized, double-blind, placebo-controlled study with a double-dummy, and crossover design. Participants were recruited via newspaper and radio advertisements, and by word of mouth. Potential candidates underwent a screening visit during which comprehensive medical and psychiatric assessments were performed to determine eligibility. Eligible individuals provided written informed consent for participation in this study. Participants were healthy adult cannabis users who were determined to be either occasional user (i.e., self-reported average cannabis use of ≥2 times per month but <3 times per week during the past 3 months) or frequent user (i.e., self-reported average cannabis use of ≥5 times per week during the past 3 months, plus a positive cannabinoids urine test at the screening visit) and were not seeking treatment for drug use. For the full list of eligibility criteria, see Appendix S1. All procedures were conducted at the National Institute on Drug Abuse (NIDA) Intramural Research Program and the Johns Hopkins Bayview Clinical Research Unit. The protocol was approved by the National Institutes of Health (NIH) Addictions Institutional Review Board (IRB), FDA, and the Drug Enforcement Administration (DEA), and was registered at ClinicalTrials.gov (NCT02177513).

**Fig. 1:**
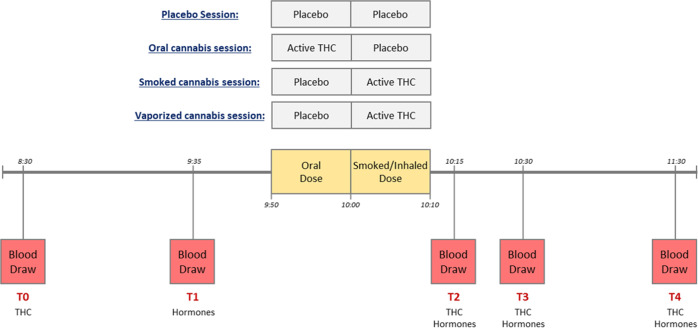
Schematic outline of the study procedures and blood sampling time-points. Each participant underwent four sessions during which placebo, oral THC, smoked THC, or vaporized THC was administred. The oral dose, either active or placebo, was always administered in the form of a brownie. Blood concentrations of total ghrelin, acyl-ghrelin, leptin, GLP-1, and insulin were assessed. GLP-1 glucagon-like peptide 1, THC tetrahydrocannabinol.

The purpose of the parent study was to examine pharmacodynamic and pharmacokinetic parameters of oral, smoked, and vaporized cannabis in occasional and frequent users; a detailed description of this study is reported elsewhere^52^. Briefly, each participant underwent four dosing sessions in a randomized order. During each session, participants received oral cannabis (or matched placebo) at 9:50 a.m., followed by smoked or vaporized cannabis (or matched placebo) at 10:00 a.m. Participants were instructed to eat, smoke, or inhale each active or placebo compound within 10 min, while they were not required to finish the dose. Only one dose of active cannabis per session was administered (Fig. 1): (A) Placebo condition: oral placebo followed by smoked or vaporized placebo; (B) Oral cannabis condition: oral cannabis followed by smoked or vaporized placebo; (C) Smoked cannabis condition: oral placebo followed by smoked cannabis; and (D) Vaporized cannabis condition: oral placebo followed by vaporized cannabis. Of note, the route of placebo administration (smoked or vaporized) was randomized across conditions A and B for each participant. The NIDA Research Technology Branch provided active cannabis and placebo for this study. Active compounds contained 6.9 ± 0.95% THC (~50.6 mg), while placebo compounds contained 0.001 ± 0.000% THC. For the active smoked dose, one 6.9% THC cigarette was administered. For the active inhaled dose, the equivalent of one 6.9% THC cigarette was ground and placed into the Volcano® Medic vaporizer (Storz & Bickel GmbH & Co, Tuttlingen, Germany). For the active oral dose, the equivalent of one 6.9% THC cigarette was ground and prepared as a brownie, using Duncan Hines® Double Fudge Brownie Mix. Placebo doses were administered with the same methodology but did not contain active ingredients. For additional details see Appendix S2.

Participants were admitted to a secure inpatient unit the night before the first dosing session to preclude acute intoxication. A standard breakfast was served at 6:00 a.m. which did not include the following food items: French dressing, mayonnaise, ketchup, raw onions, pickle relish, blue cheese, potato chips, peanut butter, butter, and margarine. The first blood draw was done at 8:30 a.m.; participants did not have any food until after the last blood draw at 11:30 a.m. (Fig. 1), when lunch was served. Nicotine smokers could smoke on the unit, but not during the 3-h experimental session. Occasional users remained on the unit 54 h post-dose and had the choice to stay or leave between sessions, as long as the dosing schedule did not exceed their self-reported cannabis use frequency. Frequent users remained on the unit 72 h post-dose and left the unit for a minimum of 72 h between sessions. These time-frames were selected based upon the window of THC detection in oral fluid from previous studies^54,55^. In order to prevent cannabis withdrawal, participants were told that they could resume their routine cannabis use between the sessions. All sessions were required to be completed within a maximum of 1 year. Cannabis administration procedures were performed consistent with the National Advisory Council on Drug Abuse Guidelines for Administration of Drugs to Human Subjects.

### Blood collection, processing, and assays

A saline lock intravenous catheter was inserted into the antecubital fossa of participants’ non-dominant arm for multiple blood samplings. For the purpose of this secondary study, blood samples were collected at five time-points during each experimental session (Fig. 1): (T0) 80 min before administration of the oral dose; (T1) 15 min before administration of the oral dose; (T2) 15 min after administration of the smoked/inhaled dose; (T3) 30 min after administration of the smoked/inhaled dose; (T4) 90 min after administration of the smoked/inhaled dose. T0 and T1 included only one blood tube for THC and hormones, respectively; T2, T3, and T4 included two blood tubes to measure both. Therefore, blood THC and hormones were each measured at four time-points during the experimental session.

For THC measurements, blood was collected in gray top potassium oxalate (8 mg)/sodium fluoride (10 mg) tubes (BD Vacutainer®), kept in an ice bath until aliquoting within 2 h into 3.6 mL Nunc® Cryotube vials (Thomas Scientific), and stored in a −20 °C freezer until analysis. A previously validated liquid chromatography–tandem mass spectrometry (LC-MS/MS) method^56^ identified and quantified cannabinoids blood concentrations. For more details, see Newmeyer et al.^52^.

For hormones measurement, blood was collected into a lavender top spray-coated K2EDTA tube (BD Vacutainer®). This tube was pre-treated with 4-(2-aminoethyl)benzenesulfonyl fluoride hydrochloride (Roche Diagnostics GmbH, Germany – Pefabloc® SC), dipeptidyl peptidase IV inhibitor (EMD Millipore Corp., Billerica, MA – Cat. #DPP4-010), and a protease inhibitor cocktail (Sigma-Aldrich Inc., Saint Louis, MO – Cat. #P8340) prior to blood collection and was inverted 10 times and kept on ice after collection. The blood tube was centrifuged within 30 min post-collection (relative centrifugal force: 1700 × *g*, temperature: 4 °C, centrifugation time: 15 min); the extracted plasma sample was pipetted into 500 µL microtubes and stored in a −80 °C freezer until analysis. All samples were run in duplicate and the assays were carried out in accordance with manufacturer’s instructions. Total ghrelin was measured with the Millipore Human Ghrelin (Total) 96-Well Plate Enzyme-Linked Immunosorbent Assay (ELISA) kit (EMD Millipore Corp., Billerica, MA – Cat. #EZGRT-89K). The optical density of each well was determined with the GloMax®-Multi Detection System (Promega Corp., Madison, WI – Part #TM297) and a regression model was applied to calculate total (i.e., acyl + des-acyl) ghrelin concentrations. The Millipore Human Metabolic Hormone Magnetic Bead Panel 96-Well Plate MILLIPLEX® _MAP_ kit (EMD Millipore Corp., Billerica, MA – Cat. #HMHEMAG-34K) was used to quantify the following analytes: active ghrelin (here referred to as acyl-ghrelin), leptin, active GLP-1 (here referred to as GLP-1), insulin, active amylin (here referred to as amylin), and Peptide YY (PYY). The assay was performed on fluorescence-coded magnetic beads coated with capture antibodies specific for each marker. Introduction of biotinylated detection antibody and streptavidin-phycoerythrin permitted simultaneous detection of all analytes on the MAGPIX® instrument (Luminex Corp., Austin, TX). These multiplex data were pre-processed and analyzed in the MILLIPLEX® Analyst software (Version 3.5 – EMD Millipore Corp., Billerica, MA) to calculate the concentration of each hormone. Values below the lower limit of quantitation (LLOQ) were set to 1/2 of the LLOQ. Amylin and PYY were removed from statistical analysis because more than 20% of the data were marked as below the LLOQ. In summary, the effect of cannabis administration on the following endocrine markers was assessed: total ghrelin, acyl-ghrelin, leptin, GLP-1, and insulin.

### Statistical methods

Participants’ demographic data were summarized with descriptive statistics (mean and standard deviation for continuous variables, number and percent for categorical variables). All data were examined for statistical outliers and normal distribution; outliers were removed, and if necessary, a logarithm transformation was applied. Leptin and GLP-1 data were not normally distributed and, therefore, were log10 transformed, which resulted in normal distribution of these data. THC pharmacokinetic parameters, including area under the blood concentration-time curve (AUC) and maximum blood concentration (Cmax), as well as baseline/pre-drug (T1) concentrations of the hormones were analyzed by analysis of variance (ANOVA) tests. Repeated measurements of endocrine markers were analyzed with linear mixed-effects (LME) models, having drug condition (placebo, oral cannabis, smoked cannabis, and vaporized cannabis), session number (1, 2, 3, and 4), blood sampling time-point (T1, T2, T3, and T4), and drug × time-point interaction as fixed effects, individual subjects as a random effect (random intercept and slope), and each hormone as the outcome. Age, gender (male, female, other), body mass index (BMI), race (Black or African-American, White or European-American, American Indian or Alaska Native, Native Hawaiian or other Pacific Islander, Asian, other), and sub-group (occasional user, frequent user) were tested as potential covariates in the initial run of each model; significant covariates were retained in the final model analysis. Pairwise comparisons between estimated marginal means of fitted models were adjusted using the Bonferroni procedure. As an exploratory outcome, we also evaluated the relationship between blood concentrations of THC and endocrine markers. To do so, AUC was calculated for each measurement across the four time-points (T0, T2, T3, T4 for THC; T1, T2, T3, and T4 for each hormone). Placebo condition was removed, as THC concentrations were steady, as expected, during the placebo sessions. Pearson’s correlation coefficients evaluated bivariate associations between THC AUC and hormones AUCs. Significant covariates in the main LME models mentioned above were included in these analyses as well. Finally, hysteresis plots provided a comprehensive visualization of the relationship between blood concentrations of THC and endocrine markers. IBM SPSS Statistics version 20.0 for Windows (Armonk, New York, USA) and GraphPad Prism version 7.0 for Windows (La Jolla, California, USA) were employed for data analysis and graphing purposes. Significance level was set at *p* < 0.05 (two-tailed) for all analyses.

## Results

### Study sample

Twenty individuals completed the study and their data were analyzed (Fig. S1). Participants were predominantly male and African-American. Demographic characteristics of the study sample are summarized in Table 1.

**Table 1:** Demographic characteristics of the study sample (*n* = 20).

Variable	Descriptive statistics
Age, years, M (SD)	28.25 (7.75)
Gender, *n* (%)	
Male	15 (75)
Female	5 (25)
Race, *n* (%)	
Black/African-American	15 (75)
White/European-American	5 (25)
Education, *n* (%)	
Some high school education	1 (5)
High school diploma	7 (35)
Some college education	9 (45)
College degree	3 (15)
Nicotine smoker, *n* (%)	
Yes	9 (45)
No	11 (55)
Weight, kg, *M* (SD)	77.21 (14.99)
BMI, kg/m^2^, *M* (SD)	25.92 (5.30)
Age at first cannabis use, years, *M* (SD)	15.60 (3.80)
Group, *n* (%)	
Occasional user	9 (45)
Frequent user	11 (55)

*BMI* body mass index, *M* mean, *n* number, *SD* standard deviation.

### THC concentrations

Blood THC concentrations (AUC and Cmax) were significantly higher under smoked cannabis, compared to both oral and vaporized conditions (Table S1). Vaporized cannabis resulted in higher blood THC concentrations than oral cannabis, but the difference did not reach statistical significance (*p* ≥ 0.05). For all participants, Tmax (i.e., the time of Cmax) was +15 min (T2) under the smoked and vaporized conditions and +90 (T4) under the oral condition.

### Endocrine markers

**Fig. 2:**
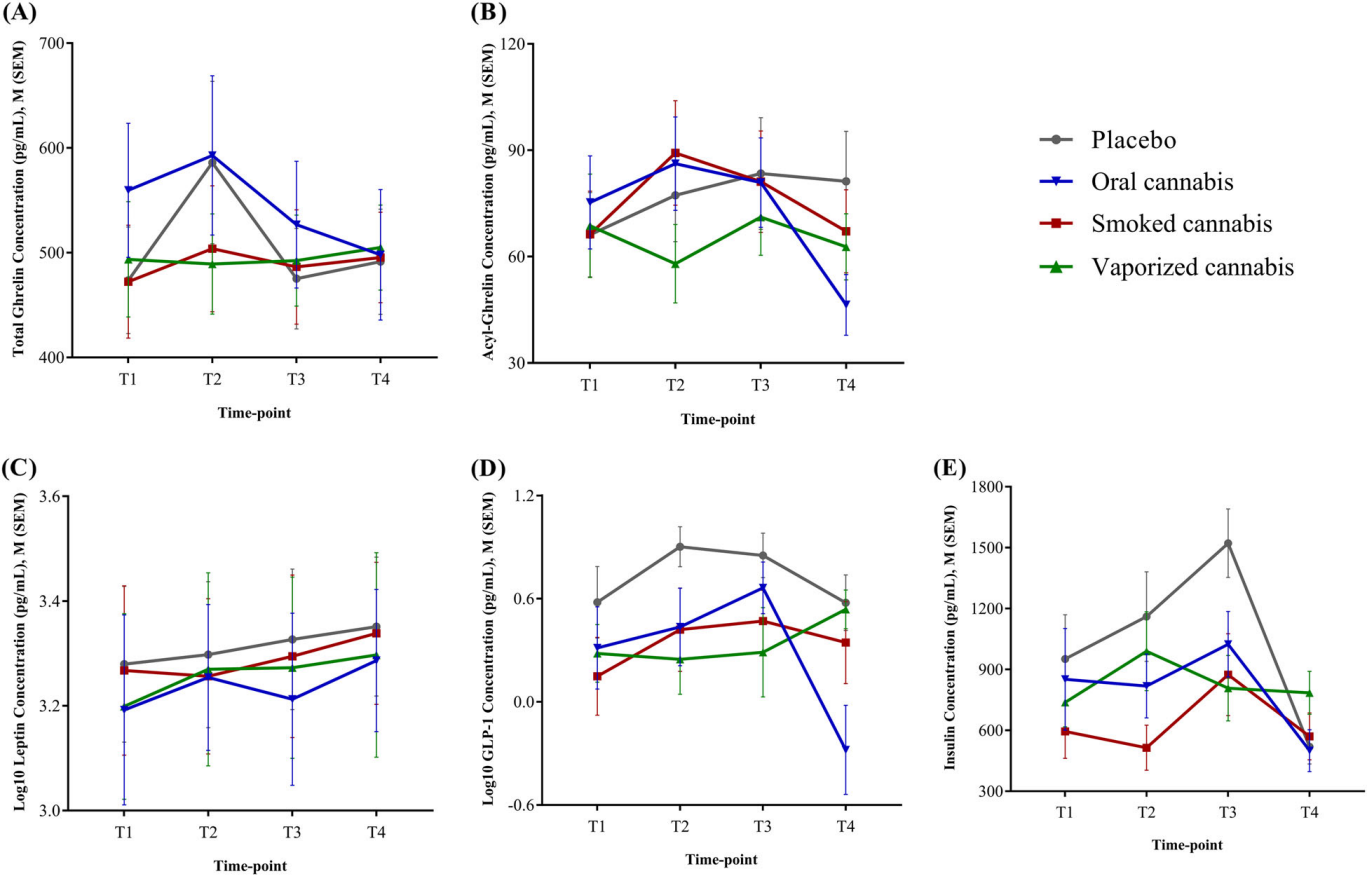
Blood concentrations of total ghrelin, acyl-ghrelin, leptin, GLP-1, and insulin during the experimental session. Blood concentrations of **a** total ghrelin, **b** acyl-ghrelin, **c** leptin, **d** GLP-1, and **e** insulin during the experimental session. For analysis results, see Table 1. For M (SEM) of each hormone per time-point per condition, see Table S3. GLP-1 glucagon-like peptide 1, M mean, SEM standard error of the mean.

Baseline concentrations of the endocrine markers at T1 were not significantly different across the four conditions (*p*’s ≥ 0.05, Table S2). The results of our main analysis (LME models) are summarized in Table 2 and graphed in Fig. 2. Briefly, a significant drug main effect (*p* = 0.001), as well as a significant drug × time-point interaction effect (*p* = 0.01), was shown for insulin, suggesting that cannabis administration blunted the increase in blood insulin concentration observed under the placebo condition (Fig. 2e). A significant drug main effect (*p* = 0.001), as well as a trend-level drug × time-point interaction effect (*p* = 0.08), was detected for GLP-1. Post-hoc analysis showed that blood GLP-1 concentrations under cannabis conditions (oral, smoked, or vaporized) were lower compared to the placebo condition. Also, a significant delayed (T4) decrease in blood GLP-1 concentration was observed under oral cannabis, but not other conditions (Fig. 2d). Finally, a significant drug main effect (*p* = 0.01) was found for total ghrelin, showing that blood total ghrelin concentrations during the oral cannabis session were higher than the smoked and vaporized cannabis sessions (Fig. 2a). No significant effects on acyl-ghrelin or leptin were found (Fig. 2b, c). Participants sub-group (i.e., occasional versus frequent user) was not a significant covariate in any of the analyses (*p*’s ≥ 0.05).

**Table 2:** Drug, time-point, and drug × time-point effects on blood concentrations of endocrine markers during the experimental session.

	Drug main effect^a^	Time-point main effect^b^	Drug × time-point interaction effect
Total Ghrelin	*F*_3,279_ = 3.44, *p* = 0.01^c^	*F*_3,279_ = 2.02, *p* = 0.11	*F*_9,279_ = 0.82, *p* = 0.59
Acyl-Ghrelin	*F*_3,279_ = 1.19, *p* = 0.31	*F*_3,279_ = 2.12, *p* = 0.09	*F*_9,279_ = 1.11, *p* = 0.34
Leptin (Log10)	*F*_3,280_ = 1.60, *p* = 0.18	*F*_3,280_ = 1.33, *p* = 0.26	*F*_9,280_ = 0.12, *p* = 0.99
GLP-1 (Log10)	*F*_3,279_ = 5.94, *p* = 0.001^d^	*F*_3,279_ = 2.09, *p* = 0.10	*F*_9,279_ = 1.70, *p* = 0.08^e^
Insulin	*F*_3,276_ = 5.50, *p* = 0.001^f^	*F*_3,276_ = 7.61, *p* < 0.001	*F*_9,276_ = 2.45, *p* = 0.01^g^

*GLP-1* glucagon-like peptide 1.

^a^Four drug conditions: placebo, oral cannabis, smoked cannabis, and vaporized cannabis.

^b^Four time-points: T1, T2, T3, T4.

Pairwise Comparison:

^c^Smoked cannabis < oral cannabis (*p* = 0.03); vaporized cannabis < oral cannabis (*p* = 0.06). See also Fig. 2a.

^d^Oral cannabis < placebo (*p* = 0.001); smoked cannabis < placebo (*p* = 0.008); vaporized cannabis < placebo (*p* = 0.01). See also Fig. 2d.

^e^T2: vaporized cannabis < placebo (*p* = 0.06); T4: oral annabis < placebo (*p* = 0.002), oral cannabis < smoked cannabis (*p* = 0.06), and oral cannabis < vaporized cannabis (*p* = 0.004); oral cannabis: T4 < T2 (*p* = 0.02) and T4 < T3 (*p* = 0.001). See also Fig. 2d.

^f^oral cannabis < placebo (*p* = 0.05); smoked cannabis < placebo (*p* < 0.001). See also Fig. 2e.

^g^T2: smoked cannabis < placebo (*p* = 0.003) and smoked cannabis < vaporized cannabis (*p* = 0.05); T3: oral cannabis < placebo (*p* = 0.06), smoked cannabis < placebo (*p* = 0.005), and vaporized cannabis < placebo (*p* = 0.001); placebo: T4 < T1 (*p* = 0.06), T4 < T2 (*p* = 0.003), T4 < T3 (*p* < 0.001), and T1 < T3 (*p* = 0.03); oral cannabis: T4 < T3 (*p* = 0.02). See also Fig. 2e.

### THC-hormones correlations

Table 3 outlines the results of correlation analyses between THC and endocrine markers AUCs. During the vaporized cannabis session, THC AUC was positively correlated with total ghrelin AUC (*r* = 0.56, *p* = 0.01); there was a trend-level positive correlation with acyl-ghrelin AUC as well (*r* = 0.40, *p* = 0.07) (Fig. S2). No other significant or trend-level correlations were found (Table 3). Hysteresis plots of the link between blood concentrations of THC and endocrine markers are demonstrated in Fig. S3.

**Table 3:** Correlation analyses between THC and endocrine markers AUCs.

	Total ghrelin AUC	Acyl-ghrelin AUC	Leptin AUC	GLP-1 AUC	Insulin AUC
THC AUC, oral cannabis session	*r* = 0.02, *p* = 0.91	*r* = 0.08, *p* = 0.71	*r* = −0.16, *p* = 0.51	*r* = −0.01, *p* = 0.96	*r* = −0.09, *p* = 0.69
THC AUC, smoked cannabis session	*r* = 0.16, *p* = 0.52	*r* = 0.08, *p* = 0.73	*r* = 0.27, *p* = 0.28	*r* = −0.07, *p* = 0.77	*r* = −0.17, *p* = 0.47
THC AUC, vaporized cannabis session	*r* = 0.56, *p* = 0.01	*r* = 0.40, *p* = 0.07	*r* = 0.06, *p* = 0.80	*r* = 0.13, *p* = 0.57	*r* = −0.20, *p* = 0.39
THC AUC, three sessions combined	*r* = 0.12, *p* = 0.35	*r* = 0.14, *p* = 0.27	*r* = 0.11, *p* = 0.39	*r* = 0.02, *p* = 0.85	*r* = −0.009*, p* = 0.94

*AUC* area under the curve, *GLP-1* glucagon-like peptide 1, *THC* tetrahydrocannabinol.

THC AUC was calculated using measurements at T0, T2, T3, and T4. Hormones AUC was calculated using measurements at T1, T2, T3, and T4. See Fig. 1.

## Discussion

To our knowledge, this study represents the first human laboratory investigation of the effects of cannabis administration, via different routes (i.e., oral, smoked, and vaporized), on peripheral concentrations of appetitive and metabolic hormones in a sample of occasional and frequent cannabis users. To summarize the key results, the most prominent influence of cannabis was on insulin, followed by GLP-1 and total ghrelin, as further discussed below.

Blood concentrations of insulin were significantly influenced by cannabis administration, as demonstrated by significant drug and drug × time-point effects. Of note, the oral dose (active THC or placebo) was always administered as a brownie. The intake of the brownie caused an expected spike in blood insulin concentrations under the placebo condition; this acute insulin spike was blunted by active cannabis administration (Fig. 2e). The effect was most evident at T3, when insulin concentrations under all THC conditions (oral, smoked, vaporized) were considerably lower than placebo. The influence of cannabis on insulin observed in this study is in line with the established role of the endocannabinoid system in regulating glucose metabolism and, at large, energy balance^57–59^. This homeostatic function is carried out via interactions between the endocannabinoid system and multiple central and peripheral pathways (e.g., brain, pancreas, liver). Through autocrine, paracrine, and endocrine mechanisms, endocannabinoids modulate pancreatic β-cells function, proliferation, and survival, as well as insulin production, secretion, and resistance^60^. Animal and human research suggest that increased activity of the endocannabinoid system may lead to insulin resistance, glucose intolerance, and obesity. Accordingly, CB_1_ receptor antagonism is associated with enhanced insulin sensitivity, improved metabolic outcomes, and weight loss^61^. It is important to note that the direction and magnitude of the relationship between cannabinoids and insulin is not linear, and may depend on multiple factors such as baseline metabolic state, duration and frequency of exposure, etc. As an example, while the aforementioned evidence suggests that overactivation of the endocannabinoid system may have negative consequences, activation of cannabinoid receptors expressed by pancreatic β-cells can stimulate insulin secretion and, therefore, may be beneficial in treating impaired glucose tolerance and diabetes mellitus^61,62^. Cannabinoid receptors are widely expressed in islets of Langerhans, and several studies have investigated specific distribution and mechanisms of CB1 versus CB2 receptors in relation to pancreatic endocrine functions^62–64^.

The majority of previous studies suggest that cannabis use acutely stimulates appetite and food intake, while chronic cannabis use reduces the risk of obesity, insulin resistance, and diabetes mellitus^25,65–68^. A recent meta-analysis on multiple replication samples found an inverse association between cannabis smoking and diabetes mellitus^24^. The evidence, however, is not strong enough to draw causal inference. Another large-scale study suggested that the beneficial impact of cannabis use on insulin resistance may not be direct, as this association was mediated through the role of cannabis in lowering BMI^69^. While insulin is primarily produced in the pancreas, feedback signals from other organs that are sensitive to cannabis may also contribute to the cross-talk between cannabis and insulin. A recent study examined whether and how different doses of THC may affect glucose uptake in the rat brain, and found that low blood THC concentrations were associated with increased glucose uptake, while high THC concentrations had an opposite effect (i.e., decreased glucose uptake)^70^. Of note, the present human laboratory study looked at acute effects of cannabis administration under a controlled condition in individuals who occasionally and frequently used cannabis. We administered a single dose of cannabis and did not have blood glucose measurements. While cannabis administration clearly suppressed the insulin spike (probably caused by the intake of brownie), the underlying mechanism of this phenomenon (e.g., direct effect on insulin production and/or secretion, interaction with glucose metabolism, or epiphenomenon) remains unknown.

Cannabis administration in the present study also modulated blood concentrations of GLP-1, an incretin closely linked to insulin and glucose metabolism. While there were no significant differences at baseline, a significant drug main effect was found, indicating that GLP-1 concentrations were lower during cannabis administration sessions (oral, smoked, and vaporized), compared to the placebo condition (Fig. 2d). These results are parallel to, and consistent with, the aforementioned effects on insulin. GLP-1 is a 30-amino acid peptide primarily produced by endocrine cells in the intestines. Upon secretion, for example in response to food intake, GLP-1 contributes to regulating blood glucose levels, mainly by stimulating insulin secretion and inhibiting glucagon secretion from the pancreas. GLP-1 also reduces food appetite and slows gastric emptying via central (e.g., hypothalamus) and peripheral (e.g., stomach) mechanisms^71,72^. The relationship between GLP-1 and insulin appears to be bidirectional, as insulin stimulates GLP-1 secretion from the enteroendocrine cells and insulin resistance, in vitro and in vivo, is associated with impaired GLP-1 secretion^73^. Hypothetically, the suppressing effect of cannabis on GLP-1 observed in this study may not be direct, but rather secondary to the suppressed insulin levels. Indeed, some of the previous research does not support a direct link between the endocannabinoid system and GLP-1 signaling. For example, increased activity of the endocannabinoid system does not influence GLP-1 concentrations^74,75^, and the effects of CB1 receptor antagonists on appetite, food intake, and weight are not mediated by GLP-1^76,77^. However, some other studies point to a possible direct link between the endocannabinoid system and GLP-1 signaling. For example, one study demonstrated that endocannabinoid-like lipids can directly bind to GLP-1 and increase its potency via conformational changes^78^. More studies are required to understand whether and how exogenous cannabis administration, as well as different components of the endocannabinoid system, may have direct and/or indirect influences on the GLP-1 system.

A significant drug main effect, but no drug × time-point effects, was found on blood total ghrelin concentrations. Post-hoc analysis showed that total ghrelin levels during the oral cannabis session were significantly higher than the smoked and vaporized cannabis sessions. Ghrelin levels during the oral cannabis session were also higher than the placebo session, but the ghrelin spike at T2 under the placebo condition may have washed out the statistical significance (Fig. 2a). In addition, a positive correlation was found between THC and total ghrelin AUCs, but only during the vaporized cannabis session (Fig. S2). As the “hunger hormone”, ghrelin plays an integral role in meal initiation, appetite, and food intake^79^. Ghrelin is also involved in glucose homeostasis, as it inhibits insulin secretion and modulates insulin sensitivity, ultimately leading to increased blood glucose levels^80,81^. The opposing physiological actions of ghrelin, compared to insulin and GLP-1, may justify the opposite direction of changes in response to cannabis in this study, i.e., increase in ghrelin levels, compared to decrease in insulin and GLP-1 levels. Growing evidence indicates a close link between the endocannabinoid system and the ghrelin system. For example, endocannabinoids and ghrelin stimulate the release and increase the activity of each other, leading to a synergistic effect^57,82^. Systemic administrations of a cannabis hydroalcoholic extract, an endocannabinoid analog, or a CB_1_ receptor agonist increased blood ghrelin concentrations in rats^83,84^. A pilot human study found a positive correlation between blood concentrations of an endocannabinoid (2-AG) and ghrelin during hedonic eating^85^. In another pilot human study, administration of smoked medicinal cannabis, compared to placebo, significantly increased blood ghrelin concentrations in HIV-infected adult men^48^. While the aforementioned findings are consistent with our results, it is hard to interpret why the effects on ghrelin in the present study were limited to specific routes of cannabis administration. It also remains unclear why the effects were specific for total ghrelin and not for acyl-ghrelin. On the latter point, it is important to keep in mind that these results are limited to the specific timeframe we studied and to the specific experimental conditions of this study (i.e., acute cannabis administration in cannabis users). Mechanistic studies are needed to disentangle the potential effects of cannabis on the synthesis, release, acylation, and/or de-acylation of ghrelin.

The results of this study should be viewed within the context of its limitations. The sample size was relatively small. As a secondary investigation, the experiment was not designed to a priori examine the outcomes presented in this report. We looked at a limited number, and not all, of the endocrine pathways involved in appetite/metabolism, and did not have measurements on other relevant biomarkers such as glucose and cholesterol levels. Given the secondary nature of this study and the number of statistical tests, we did not look at possible clinical/behavioral implications of these endocrine effects – a relevant question that was beyond the scope of the present study and should be explored in the future. Nevertheless, a comprehensive report of the subjective and physiological measures collected in this study was previously published^51^. While gender was tested as a covariate in all analyses, the small sample size and low percentage of enrolled females did not allow us to investigate possible gender differences in endocrine outcomes after cannabis administration. As a human laboratory study, the standard setting was strictly controlled before, during, and after each experiment. While such a design provides a rigorous research platform, it may not fully reflect a real-world setting. As an example, the spike in insulin levels under the placebo condition appears to be due to the intake of brownie and the effects of cannabis administration on insulin and other hormones found in this study may be dependent on this specific aspect of the design. Furthermore, only one cannabis dosage was tested, and all participants were cannabis users, without a comparison group (e.g., people with no cannabis use were not included due to ethical reasons). Therefore, our findings may not be generalizable to other feeding conditions, cannabis dosages and/or populations without further investigation.

In summary, cannabis administration, via oral, smoked, and vaporized routes, modulated blood concentrations of some appetitive and metabolic hormones in cannabis users. The most robust results of this study indicate that acute cannabis administration in cannabis users blunted the insulin spike secondary to the brownie intake. Future studies should investigate whether these findings may be replicated in larger and more diverse study samples. Understanding the mechanisms underpinning these effects is also important, as it may provide additional information on the cross-talk between cannabinoids and physiological pathways that regulate appetite and metabolism.

## Supplementary information

Spplementary Information
