# Supplementary material for: Effects of oral, smoked, and vaporized cannabis on endocrine pathways related to appetite and metabolism: a randomized, double-blind, placebo-controlled, human laboratory study
Source: Transl Psychiatry. 2020 Feb 19;10:71. doi: 10.1038/s41398-020-0756-3 (PMC7031261; doi:10.1038/s41398-020-0756-3)

**Supplementary Information**

**Appendix S1.** Study eligibility criteria

***Inclusion criteria:***

- Males and females between 18 to 50 years old
- Cannabis consumption with a minimum frequency of at least twice per month and average frequency of less than three times per week in the past 3 months (occasional cannabis user) or at least an average of five times per week in the past 3 months (frequent cannabis user)
- A positive cannabinoids urine test (only required for frequent users)
- Peripheral veins suitable for repeated venipuncture and/or placement of an intravenous catheter, as assessed by a physician’s assistant, nurse, or physician
- Systolic blood pressure ≤ 140 mm Hg, diastolic blood pressure ≤ 90 mm Hg, and heart rate ≤ 100 bpm (assessed while sitting after at least five min rest)
- Electrocardiogram (ECG) and three-minute rhythm strip without clinically relevant abnormalities
- Women with reproductive potential must either agree to abstain from heterosexual intercourse (or other means to become pregnant) or use a medically acceptable form of contraception for the duration of the study
- Must be able to safely suspend use of CNS depressant, anticholinergic, and/or sympathomimetic medications before study dosing (length of medication suspension: ≥ 3 half-lives of the medication in use)

***Exclusion criteria:***

- Current dependence on any drug other than cannabis, caffeine, or nicotine according to the Structured Clinical Interview for DSM-IV-TR Axis I Disorders (SCID)
- Current use of cannabis for medical purposes under explicit recommendation of a physician providing medical care
- Currently interested in or participating in drug abuse treatment, or participated in drug abuse treatment within 90 days preceding study enrollment
- History or presence of any clinically significant illness, as detected by history, physical examination, and/or laboratory tests, that might put the participant at increased risk of adverse events (e.g., history of psychotic disorder, clinically significant mood and/or anxiety disorder, diabetes, liver, renal or cardiovascular disease)
- Liver enzymes ≥ 2 times upper normal limit and/or clinical signs/symptoms consistent with liver disease, including but not limited to nausea, vomiting, jaundice, itching, abdominal pain, or edema
- History of clinically significant adverse events associated with cannabis intoxication (e.g., severe anxiety and panic, paranoia and psychosis, sustained tachycardia, or severe hypotension)
- History of seizures, head trauma, or other history of CNS insult that could predispose the participant to seizures
- History of food allergy or sensitivity to gluten, dairy, egg, soy, and/or chocolate
- Donation of more than 450 mL blood within 8 weeks of the first dosing session
- Hemoglobin less than 12.0 g/dL and/or clinical signs/symptoms consistent with anemia, including but not limited to fatigue, tachycardia, shortness of breath, or dizziness
- If female, pregnant or nursing
- Any form of color blindness

**Appendix S2**. Additional information about the study compounds

***Smoked cannabis/placebo:***

- Standardized cigarettes (active cannabis or placebo) were acquired from the National Institute of Drug Abuse (NIDA) Drug Supply Program (DSP). Active cannabis cigarettes (0.734±0.05 g) contained 6.9±0.95% (~50.6 mg) tetrahydrocannabinol (THC) and 0.2±0.01% (~1.5 mg) cannabidiol (CBD). Placebo cigarettes (0.713±0.05 g) contained 0.001±0.000% THC and no detectable CBD. One cigarette was used for each smoking session.

***Vaporized cannabis/placebo:***

- Volcano® Medic vaporizer (Storz & Bickel GmbH & Co, Tuttlingen, Germany) was used for vaping sessions. The content of one standardized cigarette (active cannabis or placebo) was ground to a fine powder, heated (210°C) and the vapor was collected in a plastic bag attached to the heating system for inhalation.

***Oral cannabis/placebo:***

- Brownies were prepared with Duncan Hines® Double Fudge Brownie Mix according to the manufacturer’s instructions. Equal portions of the wet batter were poured into a muffin container. The content of a standardized cigarette (active cannabis or placebo) was ground to a fine powder, placed into a greased aluminum foil packet, and baked at 121°C for 30 min to ensure decarboxylation of the acid precursor to THC. This powder was then mixed into one individual portion of brownie batter. After baking and cooling, individual brownies were stored in a freezer (-20°C) until the night before the experimental session, when they were allowed to thaw in the refrigerator (4°C) overnight.

**Figure S1**. Flow diagram of the study


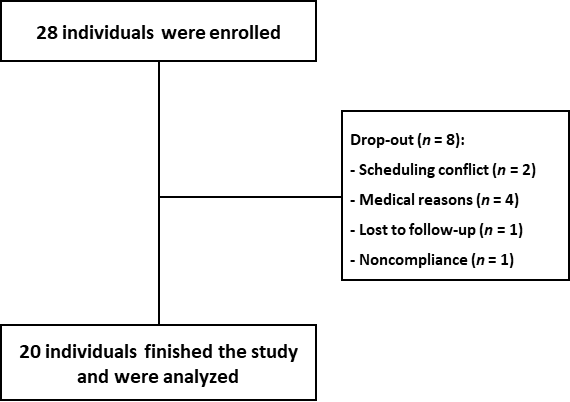


**Table S1**: Comparison of blood THC concentrations across the cannabis administration sessions^1^

| **THC Concentration** | **Session** | **M (SD)** | **One-way ANOVA result** |
| --- | --- | --- | --- |
| **AUC** | **Oral** | 476.08 (228.74) | *F_2, 56_* = 11.67, *p* < 0.001^a^ |
|  | **Smoked** | 1606.81 (1146.53) |  |
|  | **Vaporized** | 831.11 (581.61) |  |
| **Cmax** | **Oral** | 8.36 (4.25) | *F_2, 56_* = 13.17, *p* < 0.001^b^ |
|  | **Smoked** | 32.90 (23.24) |  |
|  | **Vaporized** | 16.60 (12.09) |  |

^1^ Oral cannabis, smoked cannabis, and vaporized cannabis.

^a^ Post-hoc (Bonferroni corrected):

- Smoked vs. Oral: p < 0.001
- Smoked vs. Vaporized: p = 0.006
- Vaporized vs. Smoked: p = 0.41

^b^ Post-hoc (Bonferroni corrected):

- Smoked vs. Oral: p < 0.001
- Smoked vs. Vaporized: p = 0.004
- Vaporized vs. Smoked: p = 0.27

*Abbreviations*: **ANOVA**, analysis of variance; **M**, mean; **SD**, standard deviation; **THC**, tetrahydrocannabinol.

**Table S2**: Comparison of baseline^1^ concentrations of hormones across the four study sessions^2^

| **Endocrine Marker** | **One-way ANOVA Result** |
| --- | --- |
| **Total Ghrelin** | *F_3, 76_* = 0.53, *p* = 0.66 |
| **Acyl-ghrelin** | *F_3, 76_* = 0.10, *p* = 0.95 |
| **Leptin (Log10)** | *F_3, 76_* = 0.73, *p* = 0.97 |
| **GLP-1 (Log10)** | *F_3, 76_* = 0.72, *p* = 0.54 |
| **Insulin** | *F_3, 75_* = 0.66, *p* = 0.57 |

^1^ Baseline refers to the pre-drug time-point (T1 in Figure 1 of the main manuscript).

^2^ Placebo, oral cannabis, smoked cannabis, and vaporized cannabis.

*Abbreviations*: **ANOVA**, analysis of variance; **GLP-1**, glucagon-like peptide 1.

**Table S3:** Blood concentrations of hormones at each time-point^1^ per condition.

| **Endocrine Marker** | **Time-point** | **Placebo** | **Oral**  **Cannabis** | **Smoked Cannabis** | **Vaporized Cannabis** |
| --- | --- | --- | --- | --- | --- |
| **Total Ghrelin Concentration (pg/mL), M (SEM)** | T1 | 473.65 (50.84) | 559.56 (63.98) | 472.29 (53.89) | 493.60 (55.01) |
|  | T2 | 585.99 (77.48) | 592.82 (76.06) | 503.77 (60.05) | 489.26 (47.80) |
|  | T3 | 475.23 (47.93) | 526.73 (60.45) | 486.37 (54.58) | 492.47 (43.43) |
|  | T4 | 491.39 (50.13) | 497.98 (62.26) | 495.41 (43.15) | 504.89 (40.56) |
| **Acyl-Ghrelin Concentration (pg/mL), M (SEM)** | T1 | 66.15 (11.90) | 75.28 (13.12) | 66.30 (12.14) | 68.71 (14.58) |
|  | T2 | 77.26 (13.06) | 86.22 (13.15) | 89.23 (14.73) | 57.98 (11.04) |
|  | T3 | 83.45 (15.75) | 80.88 (12.59) | 81.14 (14.29) | 71.14 (10.78) |
|  | T4 | 81.22 (14.10) | 46.39 (8.54) | 67.15 (11.69) | 62.75 (9.31) |
| **Log10 Leptin Concentration (pg/mL), M (SEM)** | T1 | 3.27 (0.14) | 3.19 (0.18) | 3.26 (0.16) | 3.19 (0.17) |
|  | T2 | 3.29 (0.13) | 3.25 (0.13) | 3.25 (0.14) | 3.26 (0.18) |
|  | T3 | 3.32 (0.13) | 3.21 (0.16) | 3.29 (0.15) | 3.27 (0.17) |
|  | T4 | 3.35 (0.13) | 3.28 (0.13) | 3.33 (0.13) | 3.29 (0.19) |
| **Log10 GLP-1 Concentration (pg/mL), M (SEM)** | T1 | 0.57 (0.20) | 0.31 (0.23) | 0.14 (0.22) | 0.28 (0.16) |
|  | T2 | 0.90 (0.11) | 0.43 (0.22) | 0.41 (0.24) | 0.24 (0.20) |
|  | T3 | 0.85 (0.12) | 0.66 (0.15) | 0.46 (0.17) | 0.28 (0.25) |
|  | T4 | 0.57 (0.16) | -0.27 (0.25) | 0.34 (0.23) | 0.53 (0.11) |
| **Insulin Concentration (pg/mL), M (SEM)** | T1 | 952.00 (217.77) | 851.81 (250.39) | 595.02 (132.63) | 737.57 (119.96) |
|  | T2 | 1160.44 (220.04) | 818.40 (157.30) | 514.61 (110.69) | 989.65 (194.29) |
|  | T3 | 1521.94 (168.74) | 1024.30 (161.59) | 874.23 (201.63) | 807.68 (160.90) |
|  | T4 | 518.88 (84.15) | 500.07 (103.01) | 570.51 (115.58) | 785.08 (105.67) |

^1^ T1: 15 minutes before administration of the oral dose; T2: 15 minutes after administration of the smoked/inhaled dose; T3: 30 minutes after administration of the smoked/inhaled dose; T4: 90 minutes after administration of the smoked/inhaled dose (see Figure 1 of the main manuscript)

**Figure S2**: Correlations between THC concentrations and (A) total ghrelin, and (B) acyl-ghrelin concentrations during the vaporized cannabis session.


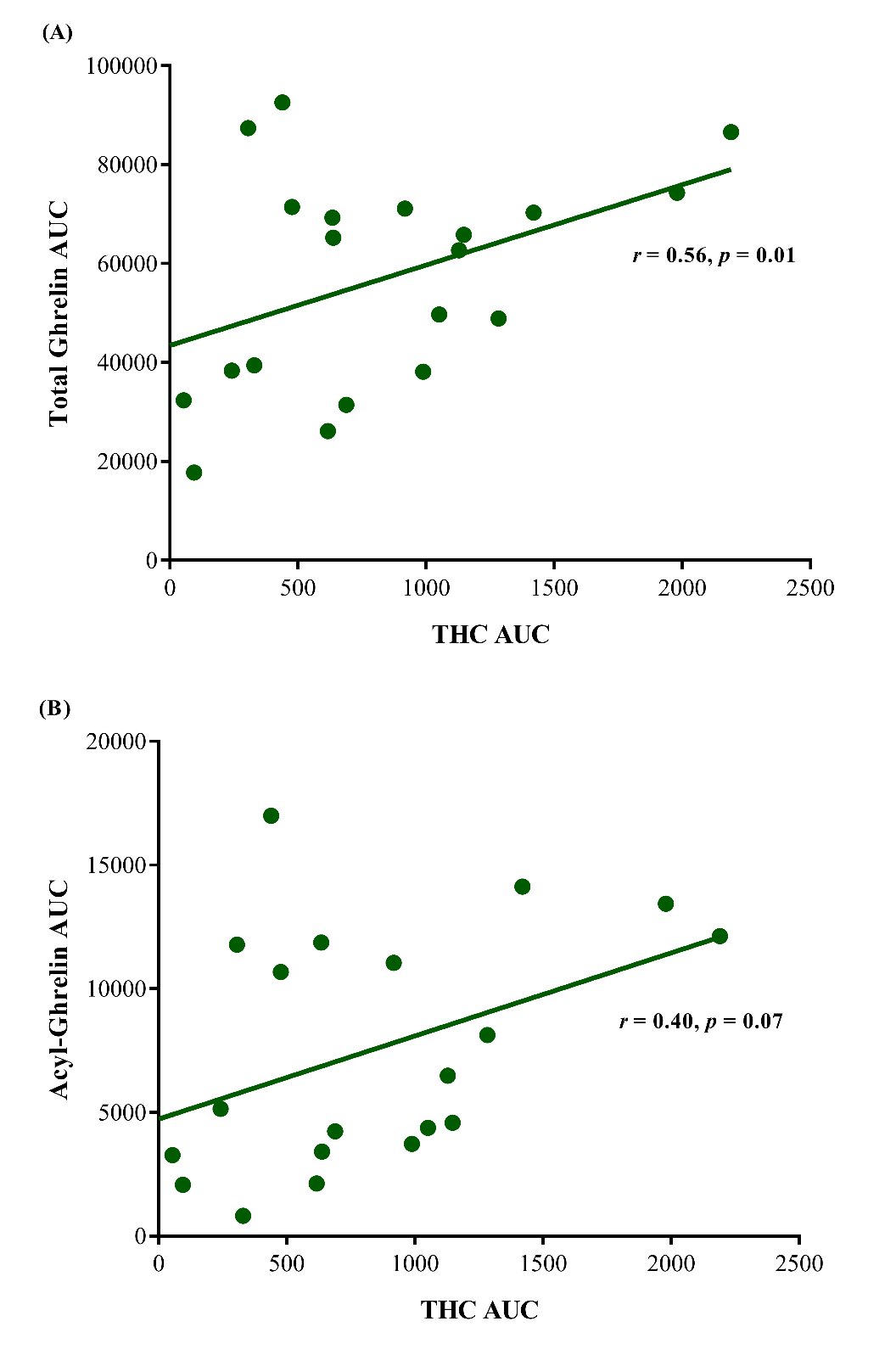


THC area under the curve (AUC) was calculated using measurements at T0, T2, T3, T4. Hormones AUC was calculated using measurements at T1, T2, T3, T4. See Figure 1 of the main paper.

**Figure S3**: THC-hormones hysteresis plots. A: first time-point (i.e., T0 for THC, T1 for hormones); B: second time-point (T2); C: third time-point (T3); D: fourth time-point (T4). See figure 1 of the main paper.


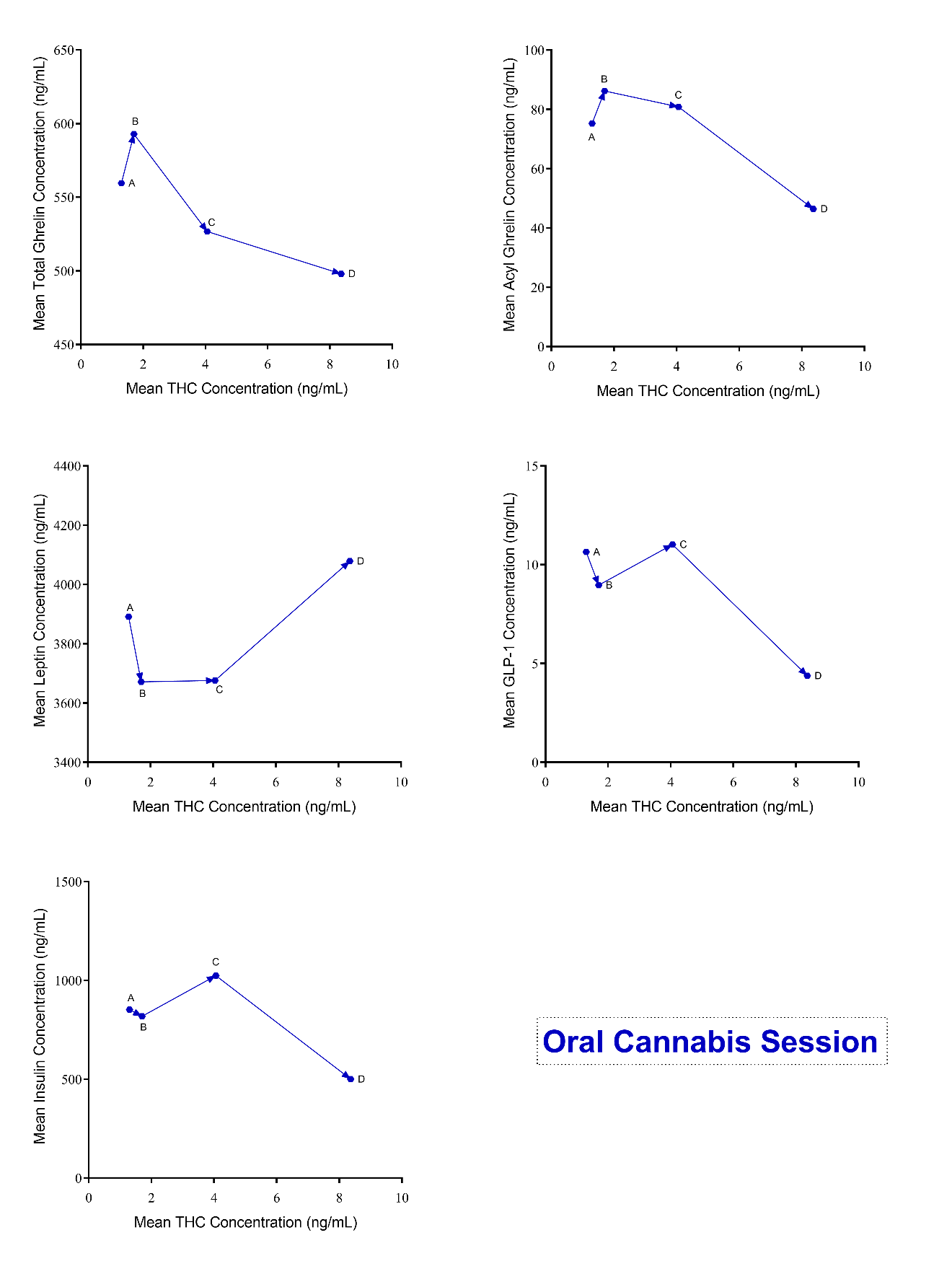


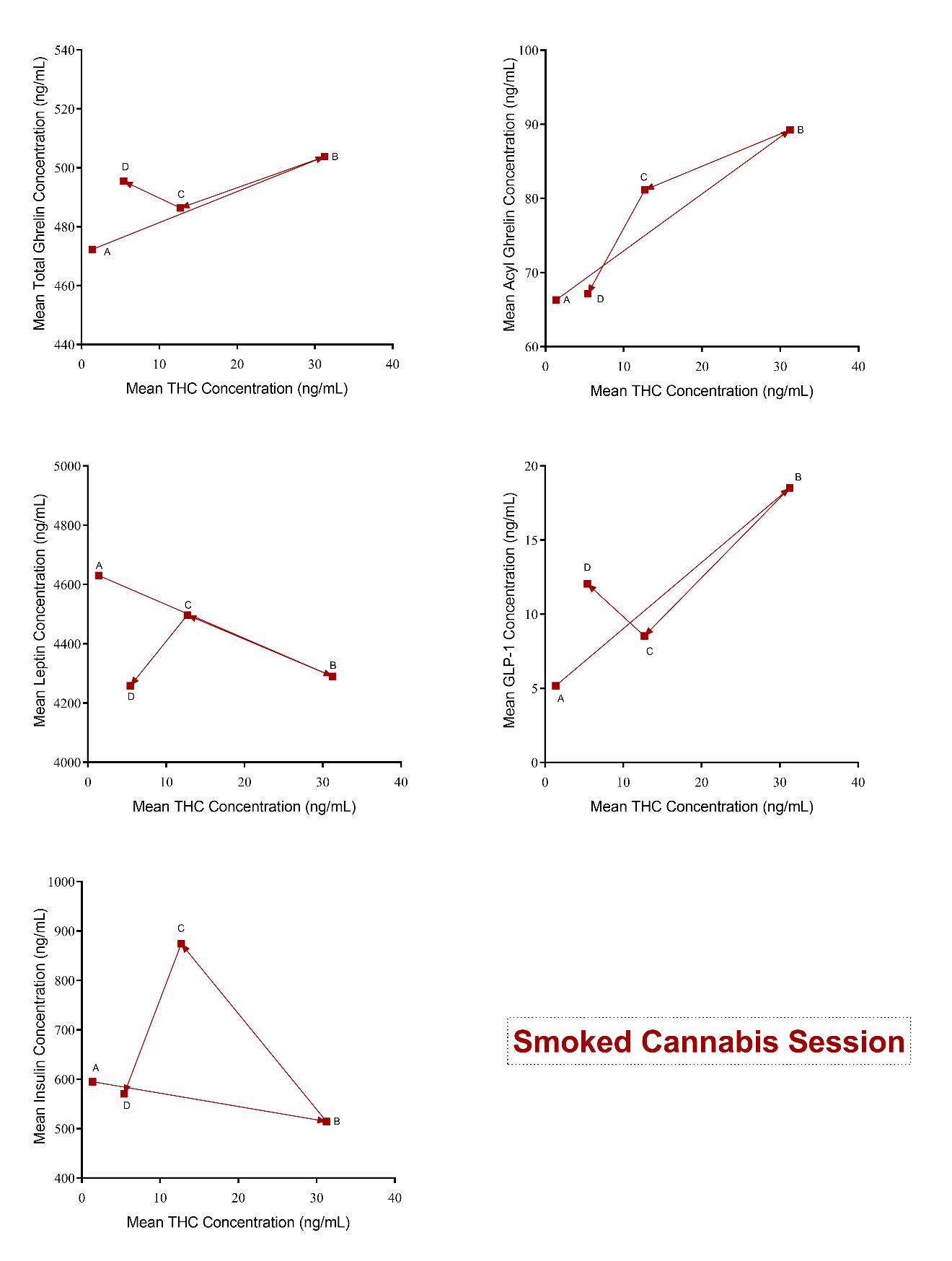


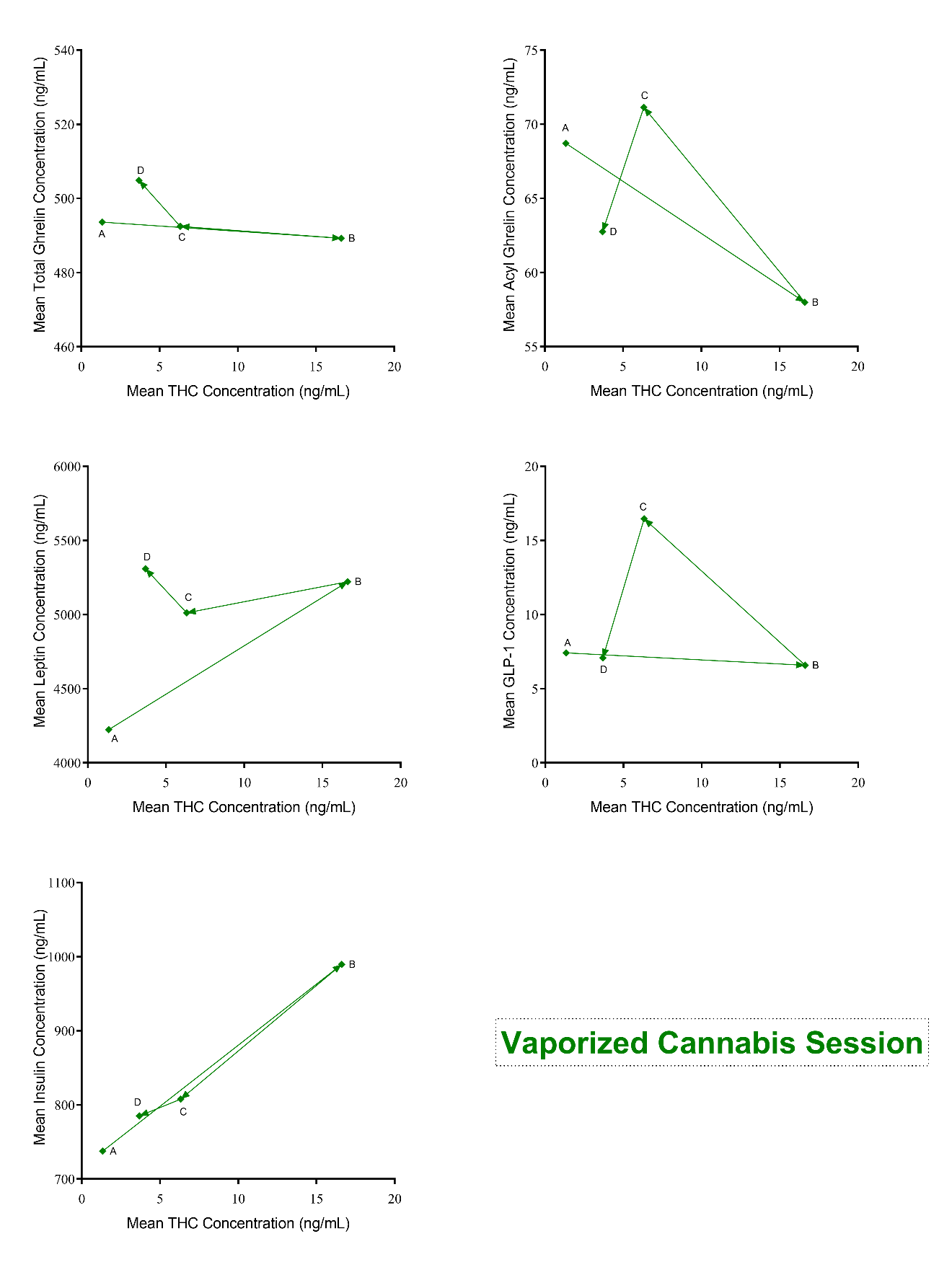

Supplement: Supplementary file 1 — Spplementary Information [file 41398_2020_756_MOESM1_ESM.docx]
